# Ulcerative Lesion Caused by Inoculation of Leishmanin

**DOI:** 10.4269/ajtmh.20-1248

**Published:** 2022-01-24

**Authors:** Jesús Rojas-Jaimes

**Affiliations:** Facultad de Ciencias de la Salud, Universidad Privada del Norte, Lima, Peru

A 16-year-old male resident of Huepetuhe (a rural area of Madre de Dios, Peru) presented with a 1-month history of a progressive, 5-cm painless skin ulcer on the back of the left hand close to the index finger. The lesion had rounded edges, seropurulent exudate, and was edematous and indurated. The patient denied previous leishmaniasis. Before microscopy was performed, a Leishmanin skin test was performed to determine whether delayed-type hypersensitivity characteristic of leishmaniasis could be elicited. Leishmanin (30 µg/mL, *Leishmania braziliensis* promastigote antigen derived from in vitro culture and produced by the Alexander von Humboldt Institute of Tropical Diseases of Universidad Peruana Cayetano Heredia in Peru) can be used to diagnose *Leishmania* infection.^1^ Positive results from microscopy or Leishmanin skin testing is sufficient to initiate drug treatment of leishmaniasis. In this patient, 0.1 mL Leishmanin was injected intradermally on the inside of the forearm. Twenty-four hours after inoculation, 7 mm of vesicular inflammation indicating a delayed-type hypersensitivity response to Leishmanin was observed at the inoculation and satellite sites. At 48 hours, the vesicle progressed to ulceration (Figure 1). Forms typical of intracellular *Leishmania* amastigotes, with visible kinetoplasts, were seen subsequently on light microscopy (×1,000 magnification) of Giemsa-stained scrapings of the primary skin ulcer on the hand (Figure 2).

**Figure 1.**
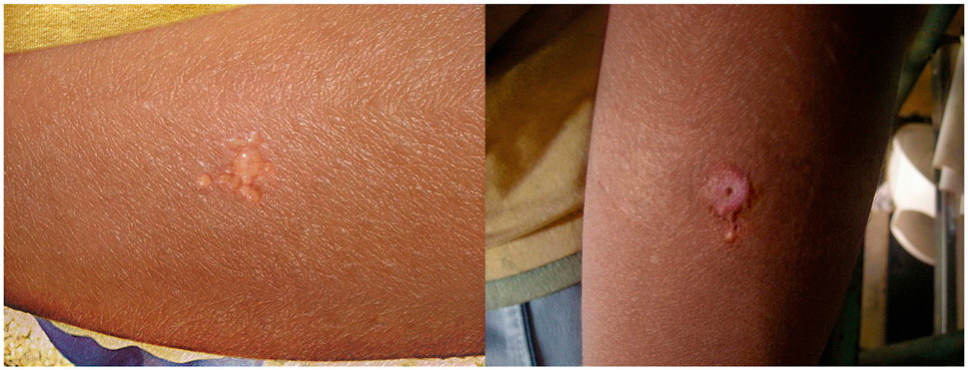
(From left to right) Inflammatory nodule 24 hours after Leishmanin injection and reaction 48 hours later. This figure appears in color at www.ajtmh.org.

**Figure 2.**
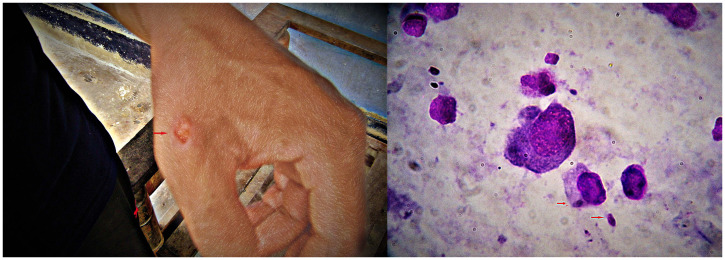
(From left to right) Primary lesion on the back of the left hand close to the index finger, and light microscopy demonstrating amastigotes (×1,000 magnification). This figure appears in color at www.ajtmh.org

The skin response to Leishmanin indicates a delayed-type hypersensitivity, T-cell-mediated immune response to *Leishmania* parasites, which corresponds to ulcer formation and is consistent with *Leishmania* species infection.^2^ The Leishman skin test cannot be considered specific for any *Leishmania* species.^3^ It is important to identify the infecting *Leishmania* species because clinical complications and treatment are species specific, such as mucocutaneous infection resulting from *L. braziliensis* (found in the Madre de Dios region), which has the potential to disseminate to the upper respiratory tract and may be difficult to treat with standard regimens aimed at cutaneous leishmaniasis-causing species.

Leishmaniasis presents with varied clinical manifestations, including cutaneous, mucosal, and visceral, that are treated with antimonials or other agents that have adverse effects.^2^ The evolution of the disease is determined by a combination of parasite species and the host response.

## References

[1] Boggild AK Ramos AP Espinosa D Valencia BM Veland N Miranda-Verastegui C Arevalo J Low DE Llanos-Cuentas A , 2010. Clinical and demographic stratification of test performance: a pooled analysis of five laboratory diagnostic methods for American cutaneous leishmaniasis. Am J Trop Med Hyg 83: 345–350. 20682880 10.4269/ajtmh.2010.09-0414 PMC2911183

[2] Maurer-Cecchini A 2009. Immunological determinants of clinical outcome in Peruvian patients with tegumentary leishmaniasis treated with pentavalent antimonials. Infect Immunol 77: 2022–2029. 19237520 10.1128/IAI.01513-08 PMC2681755

[3] Carstens-Kass J Paulini K Lypaczewski P Matlashewski G , 2021. A review of the Leishmanin skin test: a neglected test for a neglected disease. PLoS Negl Trop Dis 15: e0009531. 34292942 10.1371/journal.pntd.0009531 PMC8297750

